# Assess the feasibility of flipped classroom pedagogy in undergraduate nursing education in Sri Lanka: A mixed-methods study

**DOI:** 10.1371/journal.pone.0259003

**Published:** 2021-11-05

**Authors:** Punithalingam Youhasan, Yan Chen, Mataroria Lyndon, Marcus A. Henning

**Affiliations:** 1 Centre for Medical and Health Sciences Education, Faculty of Medical and Health Sciences, The University of Auckland, Auckland, New Zealand; 2 Department of Medical Education & Research, Faculty of Health-Care Sciences, Eastern University, Batticaloa, Sri Lanka; National Taiwan University of Science and Technology, TAIWAN

## Abstract

**Background:**

The nursing education system has evolved with an increased emphasis on student-centred education, such as implementing flipped classroom pedagogy. Given the promising positive educational outcomes, the trend of using flipped classroom pedagogy has become increasingly popular in undergraduate nursing education. However, little is known about how these flipped classroom methods impact on nursing educational practices in limited-resource settings situated in South Asia.

**Objective:**

To assess the feasibility of implementing flipped classroom pedagogy in undergraduate nursing education from the nursing students’ perspective.

**Methods:**

This mixed-methods study employed a quantitative survey and six focus group discussions conducted in three state universities in Sri Lanka. The Nursing Students’ Readiness for Flipped Classroom (NSR-FC) questionnaire was used to collect quantitative data. The semi-structured focus group discussions were conducted by using 18 reflective and open-ended questions. Descriptive statistics and multivariate analysis of variance methods were employed when analysing quantitative data. An inductive thematic analysis approach was used to summarize the focus group discussions.

**Results:**

The questionnaire survey revealed that nursing students reported high levels of personal, technical, and pedagogical readiness across all three universities, while environmental readiness was perceived as low. The inductive thematic analysis identified three themes, namely: enablers, challenges, and benefits. Specifically, nursing students valued the student-centred approach. They were ready to utilize their own devices to overcome limited technological provision; however, a short training session about how to engage in the flipped classroom was desirable. Also, their exposure to basic educational technology was perceived as adequate and they were aware of the positive outcomes of flipped classroom pedagogy.

**Conclusion:**

Nursing students were ready to enrol in a flipped classroom programme. The provision of technological resources in the education environment was identified as a great challenge for flipped classroom implementation. Overall, the findings indicate there are promising feasibilities for the flipped classroom implementation.

## Introduction

**Fig 1:**
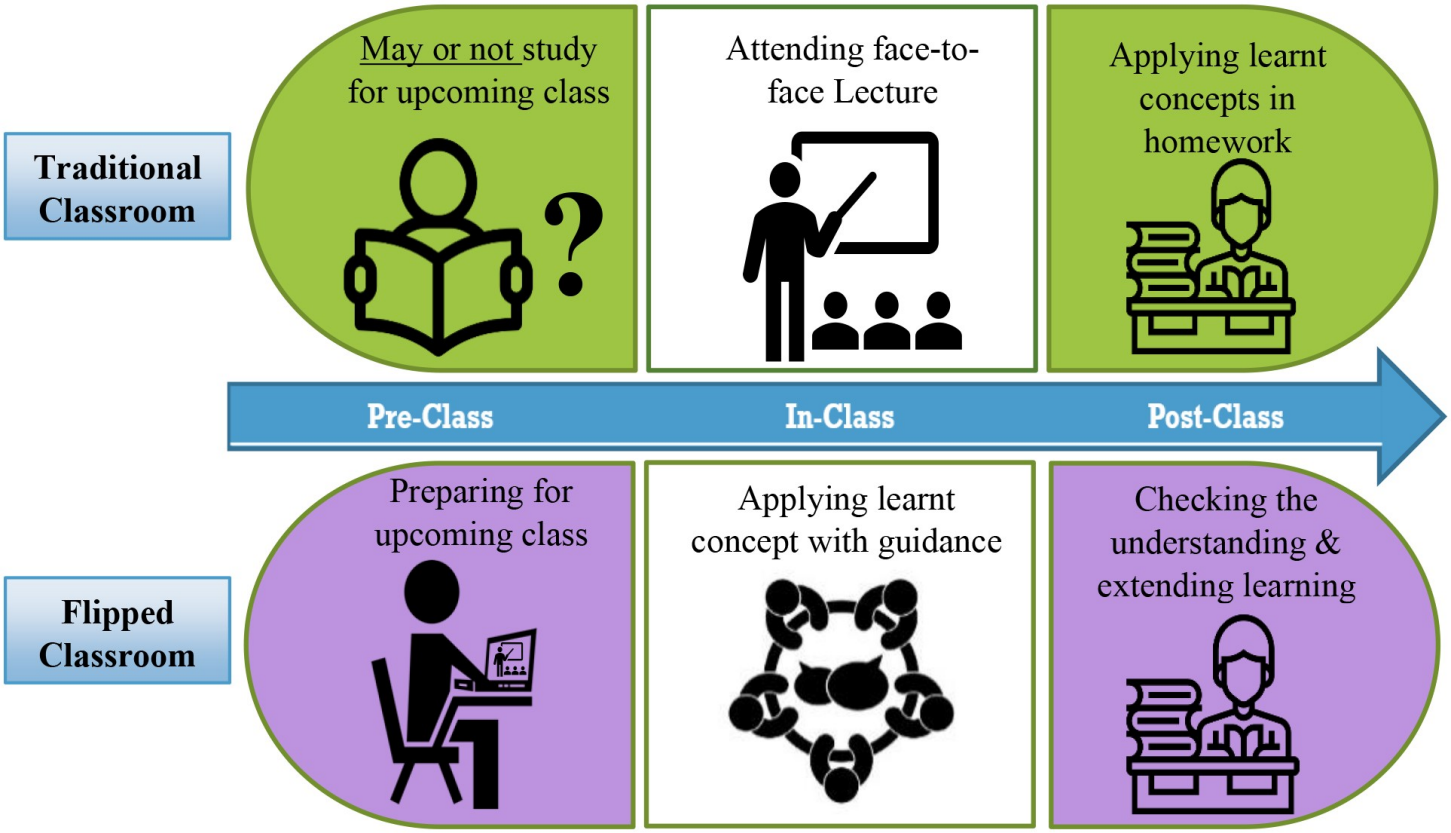
Feature of flipped classroom and traditional classroom.

The flipped classroom is an innovative active learning strategy [1]. It is a form of blended learning, including online and face-to-face teaching-learning components [2]. The flipped classroom can be implemented in three phases: pre-class, in-class, and post-class [3, 4]. Pre- and post-class are conducted online, and in-class is designed as face-to-face teaching [4]. The flipped classroom is different from the traditional classroom [5]. In a traditional classroom, nursing students are typically exposed to new knowledge during the face-to-face session, followed by take-home tasks which require students to apply the learned knowledge in their own environment with minimal support [6]. In a flipped classroom, nursing students attend a face-to-face classroom with pre-existing knowledge, which was gained through pre-classroom learning activities. Teachers design face-to-face classrooms, aligned with the flipped classroom, using student-centred learning methods and extend learning through post-class activities by using online learning resources (Fig 1) [3, 7]. The flipped classroom enables nursing students to be exposed to pre-existing knowledge applications, which instil high ordered thinking practices. This higher ordered thinking adds to face-to-face teaching and the amalgamated approach ensures that nursing graduates are experiencing deeper learning and, thus, more proficient in terms of meeting their nursing core competencies. In addition, nursing students are likely more entrustable in providing high quality and safe care to patients [8].

The current literature suggests that flipped classroom pedagogy generates numerous benefits for nursing students; it fosters their self-directed learning [9]; promotes critical thinking [1]; increases clinical performance [10, 11]; improves problem-solving skills [12]; enhances students’ therapeutic communication [11]; improves students’ engagement [13]; promotes the motivation to learn [14]; and increases assessment performance [15]. Thus, flipped classrooms can increase the potential for applying learnt knowledge into clinical contexts when implemented in the undergraduate nursing curriculum. A recent review reported that flipped classrooms had become a widely popular teaching pedagogy in nursing education in well-resourced settings [3]. Nevertheless, flipped classroom implementation is still in its infancy in low-resource settings, like South Asian Universities. Therefore, this study was designed to assess the feasibility of implementing flipped classroom pedagogy in three state universities in Sri Lanka.

**Fig 2:**
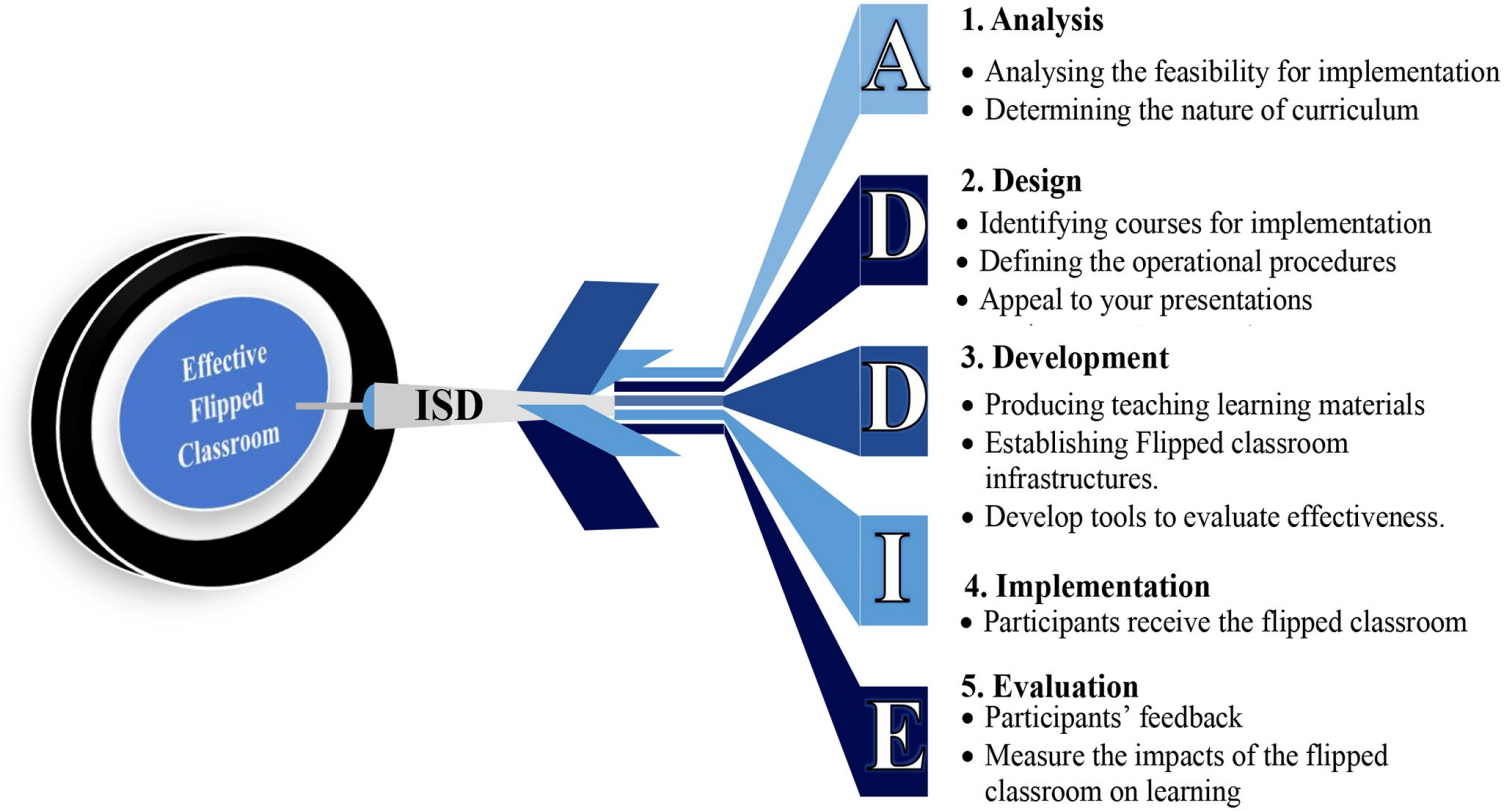
Use of the ADDIE concept when designing flipped classroom pedagogy.

The systematic process of planning and implementing a new pedagogy is called Instructional System Design (ISD) [16]. A commonly used tool which incorporates an instructional system design is “ADDIE” [17]. The ADDIE acronym stands for Analyse, Design, Develop, Implement, and Evaluate. As mentioned in Fig 2, the analyse phase focuses on assessing curriculum and feasibilities with respect to implementing flipped classroom pedagogy in nursing education. The design phase involves identifying nursing courses or modules for implementation, defining the operational procedures, and designing presentations. The development phase deals with producing teaching-learning materials, establishing flipped classroom infrastructures and developing tools to evaluate the effectiveness of the flipped classroom intervention. Nursing students receive flipped classroom experience in the implementation phase. The evaluation phase is concerned with assessing the effectiveness of the flipped classroom practice [3, 15]. According to the ADDIE tool, assessing feasibility is the first step when implementing flipped classroom pedagogy in a new education environment (Fig 2). Oh et al. (2019) proposed that assessing nursing students’ perception is indispensable in the analysis phase to align the compatibility of flipped classroom pedagogy with the defined educational context [15]. Students’ readiness, needs, and agreements with flipped pedagogy are the critical pillars to ensure its successful implementation. Student’s readiness implies they have cognitive and physical preparedness when enrolling in a flipped classroom [7]. As aforementioned, this study specifically aimed to investigate the first step (Analyse) of the ADDIE model by answering a research question regarding what is the feasibility for implementing flipped classroom pedagogy in the context of undergraduate nursing education in Sri Lanka from the perspective of undergraduate nursing students.

## Materials and methods

### Study design and setting

A mixed-methods study design, consisting of a questionnaire survey and focus group discussions, was used to assess the feasibility for implementing flipped classroom pedagogy in undergraduate nursing education in Sri Lanka. The following three state universities participated in the study: The University of Colombo (University-A), the Eastern University, Sri Lanka (University-B), and the University of Peradeniya (University-C). The three universities offer free four-year undergraduate nursing education (Bachelor of Science Nursing) which is funded by the University Grants Commission through the Sri Lankan Government. In accordance with the available reports, the Sri Lankan government invested 2.12% of its Gross Domestic Products (GDP) for education [18], which was approximately half of the mean value for educational investment (4.0%) in reference to lower-middle-income countries [19].

### Questionnaire survey

All nursing students (N = 506) from the three universities were invited to participate in the questionnaire survey. Nursing Students’ Readiness for Flipped Classroom (NSR-FC) questionnaire was used in this anonymous quantitative survey. The NSR-FC is an acceptable measurement scale for assessing nursing students’ readiness for the flipped classroom in terms of its construct validity (standardized root mean square residual = 0.08, root mean square error of approximation = 0.08, comparative fit index = 0.87, and χ2/degrees of freedom = 1.57) and reliability (Cronbach α = 0.9) [7]. The NSR-FC is a 20-item instrument consisting of four factors: personal readiness, technological readiness, environmental readiness, and pedagogical readiness. Responses were rated by a five-point Likert scale with responses ranging from 1 (Strongly disagree) to 5 (Strongly agree). The survey was distributed after a scheduled class time. Participants were clearly informed that the voluntary return of the questionnaire to the collection box indicated their consent to participate in the anonymized survey. Descriptive statistics were computed to measure student’s readiness for each item in the NSR-FC questionnaire. Mean readiness scores were calculated for each factor in the NSR-FC. Multivariate analysis of variance methods and a Bonferroni post hoc analysis was conducted to evaluate response differences amongst the three universities. All the statistical analyses were performed using SPSS ver.26 (IBM Corp., Armonk, NY, USA).

### Focus group discussion

Open invitations were given to all nursing students to participate in the focus group discussions (FGD) at the end of the questionnaire survey. The semi-structured focus group discussions consisted of 18 reflective and open-ended questions (S1 File). After reviewing a thesis [20], the authors developed the set of questions that aimed to elicit nursing students’ opinions about opportunities and challenges for implementing flipped classrooms and their willingness to enrol in a flipped classroom. One author (PY) facilitated the face-to-face FGDs. The FGDs were extended from 45 minutes to one hour. All FGDs were conducted using the English language. FGDs were recorded digitally and transcribed verbatim. Transcripts of the FGDs were analysed using NVivo [21, 22]. In accordance with the guidelines described by Thomas [23], the general inductive thematic analysis was performed to analyse the FGD data. First, open coding of all FGDs was performed to classify recurring topics. Open coding required that single sentences or phrases were identified as a code (a defined unit of language) that represented the succinct meaning of the text. Next, the codes were aggregated into themes (broader definitions). The initial coding was done by two authors (PY and YC) after multiple readings and interpretation. The coding was cross-checked by the other two authors (ML and MAH). The process of assigning codes into themes was performed in meetings with the presence of all four authors. Accordingly, the authors met many times to validate the themes until consensus was reached. Then, the relationship between themes were identified. PY has taken a significant role in interpreting the qualitative findings. He has formal teaching experience in undergraduate nursing education in Sri Lanka. He was able to discuss his positioning and reflections with other authors to ensure that his process was not prejudiced. Therefore, the trustworthiness of inductive analysis was verified by three authors (YC, ML and MAH) through auditing the rigour and authenticity of the data classification process.

### Ethics approval and consent to participate

The study was approved by the University of Auckland Human Participants Ethics Committee (Reference Number 024079). Participants’ information sheets were provided before administering the anonymized questionnaire and starting the FGDs. Approvals for collecting data were obtained from relevant heads of the selected universities. Study data and recordings were treated with strict confidentiality. A confidentiality agreement was signed by the transcriber, and the transcriptions were anonymized.

## Results

### Characteristics of the participants

The Table 1 below gives information about the methods used to collect data, and the characteristics of the participants (in terms of gender and academic year of study) in the three Sri Lankan universities. In total, 396 undergraduate nursing students responded to both the survey and engaged in the FGDs (i.e., Questionnaire survey [n = 355] and FGDs [n = 41]). More specifically, two hundred and fifty-six females and 99 males took part in the questionnaire survey (university-A [n = 141], university-B [n = 99], and university-C [n = 115]). The response rate was 70.15%, which reasonable response rate for a questionnaire survey [24]. The mean age of the participants was 23.35 years. Six focus group discussions (FGDs) were conducted, two FGDs were conducted in each university (For example, FGD-1 & FGD-2 were held at the University-A). Each FGD included six to nine students.

**Table 1:** Characteristics of the study participants who attend focus group discussion and questionnaire survey.

University	Survey Method		Sample Size	Gender		Academic Year of Study
				Male	Female	
**University-A**	Focus Group Discussion	FGD-1	9	4	5	2^nd^
		FGD-2	7	3	4	1^st^
	Questionnaire Survey		141	41	100	1^st^ (n = 78), 2^nd^ (n = 63)
**University-B**	Focus Group Discussion	FGD-3	6	2	4	3^rd^
		FGD-4	7	5	2	4^th^
	Questionnaire Survey		99	30	69	1^st^ (n = 23), 2^nd^ (n = 28), 3^rd^ (n = 28), 4^th^ (n = 20)
**University-C**	Focus Group Discussion	FGD-5	6	1	5	2^nd^
		FGD-6	6	3	3	3^rd^
	Questionnaire Survey		115	28	87	1^st^ (n = 38), 2^nd^ (n = 35), 3^rd^ (n = 23), 4^th^ (n = 19)

Note: FGD, Focus Group Discussion.

### Quantitative findings from questionnaire survey

Table 2 provides detailed information obtained from nursing students regarding their readiness for engaging in a flipped classroom. In Table 2, details are split into several columns. The first column gives the factor details of NSR-FC. The items embedded within each NSR-FC factor are listed in the second column. The third column describes the overall percentage of students’ perceptions. The fourth column explains the mean perception for NSR-FC factors at the university level. The last two columns show F- and P-values for NSR-FC factors in relation to difference between the three universities.

**Table 2:** Nursing student’s readiness for flipped classroom.

Factor	Statement	Overall students’ perception (in %)					Mean perception at university level				F-Value	P-Value
		SD	D	U	A	SA	UA	UB	UC	Overall		
**Personal Readiness**	I am willing to engage in flipped learning.	0.85	0.28	8.17	33.80	56.90	4.38	4.05	4.22	**4.21**	10.08	0.000
	I am willing to make the time available for flipped learning.	0.28	2.54	9.01	48.17	40.00						
	I am interested in achieving my learning outcome through flipped learning.	0.56	1.41	5.35	39.72	52.96						
	I need hands-on training for engaging in a flipped classroom.	0.56	4.79	20.28	47.32	27.04						
	I am interested in playing online quizzes as a classroom activity	2.25	5.35	13.52	40.00	38.87						
**Technological Readiness**	I can use document viewing software (i.e., Adobe Reader) to read materials.	1.13	4.51	6.48	35.77	52.11	4.30	4.20	4.49	**4.33**	6.59	0.002
	I can use instant messaging software (i.e., Viber, WhatsApp, Skype and Twitter) to communicate with people.	1.41	2.25	4.51	32.39	59.44						
	I can download files from the internet.	2.54	3.38	9.30	34.08	50.70						
	I can operate online media players (i.e., VLC Media Player) to watch or listen to multimedia materials.	1.13	4.79	12.39	34.37	47.32						
	I can search for the information that I need from online resources.	1.41	2.54	4.51	28.73	62.82						
	It is convenient for me to use a computer and or mobile phone in my learning.	1.13	2.25	8.17	37.18	51.27						
	I am familiar with learning from video lectures (e.g. in YouTube).	1.69	3.10	12.96	37.75	44.51						
**Environmental Readiness**	I have access to the internet connection in the University (E.g. WiFi).	52.11	8.45	6.20	16.62	16.62	1.65	2.44	4.05	**2.71**	320.34	0.000
	My university provides the necessary resources for flipped learning.	32.11	20.56	13.80	25.63	7.89						
	My university promotes technology-enhanced learning practices among students	31.55	17.75	15.21	25.63	9.86						
	Technical help is available for e-learners in the university.	29.58	18.03	12.68	24.23	15.49						
	Computer labs in my institutions are the most important assets for using flipped learning.	28.17	14.08	14.37	28.73	14.65						
**Pedagogical Readiness**	I prefer a student-teacher interaction at an individual basis (1:1) to clarify doubts.	2.25	3.66	14.93	44.51	34.65	4.15	3.86	4.21	**4.07**	7.37	0.001
	It would be convenient if an online platform could be used to interact with teachers and classmates.	1.97	1.97	13.52	48.45	34.08						
	I prefer a student-centered classroom learning process (such as role-play, problem-based learning, debates and quizzes) rather than learning from a traditional lecture.	1.97	3.38	14.93	41.41	38.31						

Notes:

1. Overall students’ perception anchors are denoted as: SD, Strongly Disagree; D, Disagree; U, Undecided; A, Agree; SA, Strongly Agree.

2. University abbreviations are denoted as: UA, University A (University of Colombo); UB, University B (Eastern University, Sri Lanka); UC, University (University of Peradeniya).

Overall, the findings (Table 2) indicate that nursing students’ scores were skewed towards high scores from all three universities for the domains of personal readiness (4.21), technical readiness (4.33) and pedagogical readiness (4.07). However, lower levels of skewness were generated for environmental readiness (2.71).

The multivariate test analysis, using the Wilks’ Lambda test, showed significant effects were evident (Wilks’ Lambda = 0.33, *F* (8, 698) = 64.74, *p* < .001). The between-subjects test showed significant differences between the three universities for all domain measures (see Table 2). The Bonferroni post-hoc analysis revealed specific differences between universities. Firstly, the university scores for the personal readiness domain revealed that the only significant difference was that university-A (4.38) generated higher scores than university-B (4.05). Secondly, the scores for the technological readiness domain showed that significant differences were evident between university-C (4.49), which generated higher scores than universities A (4.30) and B (4.20). Thirdly, the scores for the environmental readiness domain showed that university-A (1.65) generated significantly lower scores than universities B (2.44) and C (4.05), and university-B generated lower scores than university-C. Lastly, the scores for the pedagogical readiness domain showed that significant differences were evident between university-B (3.86), which generated lower scores than universities A (4.15) and C (4.21). No other significant differences were noted.

### Qualitative findings from focus group discussions

**Fig 3:**
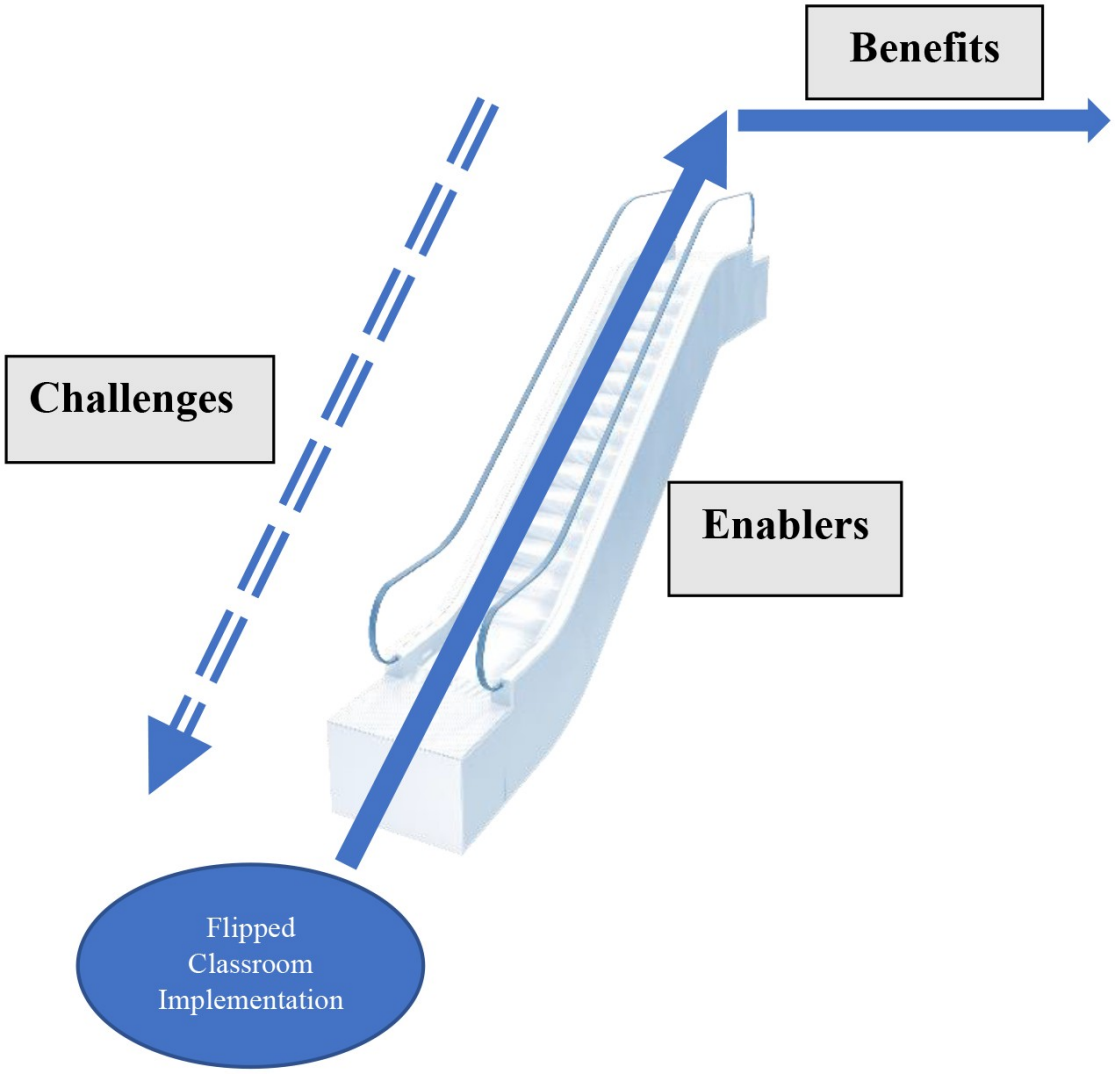
Themes and their relationship.

The current inductive analysis categorised the focus group discussion into three themes, namely: enablers, challenges, and benefits. The themes, sub themes and sample quotes are listed in Table 3. Using Thomas’ method [23], we developed a model that accurately represented the connections between the emerging themes, and this was consistent with the perceived underlying structure of students’ perceived experiences regarding the flipped classroom pedagogy and these experiences were evident within the transcribed text. Therefore, the relationship between themes were identified (Fig 3).

**Table 3:** Qualitative themes with exemplar codes and quotes.

Themes	Sub themes	Sample Codes and Quotes
Enablers	Readiness	Open to try flipped classroom *We are ready to face the flipped classroom* *I think now is time to change the learning method* *We are ready to receive online teaching* Pre-class learning *If the lecturers ask us to do pre-learning*, *we will do…* *We will be more prepared*, *and we know what the lecturer is going to talk* *Preparing ourselves before we go to the lecture will stimulate our thinking*
	Use of technology and available support	Device and online apps *Ready to use laptops*, *phones*, *and YouTube* *We all afford to use our private devices for learning* *We can manage it (i*.*e*., *flipped classroom) with our smartphones* *We are using mobile phones and laptops to access the Moodle* *We all have Moodle in our smartphones* *I am using technological devices which more helpful and interesting in the studies than reading books or papers* Technical support *If we have any problem with technology*, *we can call a special technical team here at any time* *We have Wi-Fi facilities in our university*. *So*, *we can use it only for studying purposes* *If we start first*, *I guess the university will provide the resources*
	Current student-centred teaching learning practices	Current face-to-face teaching practices *We followed a problem-based learning approach*, *it helped me improve critical thinking and clinical skills* *We engaged with students’ base activities like students’ presentation* *Currently we are using this type of method (flipped classroom) in some lectures* Ways to clarify doubts *If we have any doubts*, *we can ask them during the session* *Lecturers allow us to talk with others in the lecture time if we have any confusions*. Sharing teaching materials with students *Some of the lecturers are giving presentations as soft copy* *Lecturers sometimes put their notes in the Moodle too* Current pre and post-class activities *Some lecturers give information about upcoming session* *They (teachers) sent some basic questions for our preparation* *Most of the time after the lecture they (teachers) gave some assessment and assignment* *Sometime the lecturers give some documents to study after the lecture*. Self-directed learning *Teacher gives basic information that is not enough*. *We have to search advanced things* *I go through YouTube videos if I have any doubts*
Challenges	Limited infrastructure	Connectivity *If we have free Wi-Fi*, *it will be better* *When all the students are using Wi-Fi*, *it will be slow down* Limited Support *We did not get any technical support*. *Our faculty is recently started in this University*, *I think we will get support in the near future*
	Student motivation	Inactive learning *We are like lazy in the lecture* *Sometimes we do not know what they are trying to teach because we are just passively listening only* *Most of the student’s mentality focuses on 80% attendance”*. Classroom Management *Teachers are being like authoritarian*, *like in school* *There is no individual attention*.
	Lack of pre-class learning	No pre-learning *I’m going to lecture blindly* *I never prepare before the lecture* *We do not get the next day’s topic the day before*
	Training and development	Training needs *Training should need for enrolling this (Flipped classroom)* *We need to develop some soft skills to concentrate & engage in classroom activities* *Lecturers should have more knowledge about flipped classroom because they are going to guide us* Development *The video should be attractive* *Just only watching (Video Lecture) is difficult* *We have to find some mechanism to see the video before coming to the face-to-face lecture*
Benefits	Positive educational outcomes	Educational outcomes *I think we get more engaged* *It will keep us more energetic & active* *It will make us more attentive* *In the flipped classroom*, *students will be more confident to talk*
	Knowledge transfer & application	Applying knowledge *In nursing*, *theory is not that much enough so we have to practice & apply* *In the flipped classroom*, *we will get the opportunity during the lecture time to apply the things what we learned* *A normal classroom with teacher-based lectures are not help us to deal with patients*
	Flexible time management	Impact on time *Flipped classroom will positively impact on time* *It will decrease our time wastage* Utilizing free time *In the flipped classroom method*, *we can watch the video lecture during our free time* *We are spending more hours on social media so we can use that time to watching video lectures*
	Flexible audio-visual material	Effectiveness of video lecture *Video lecture is effective* *Easy to remember* *Easy to understand* *We can watch that according to our speed* *Learning through books is boring*. *if it is video*, *we can watch it quickly*
	Enhanced teacher-student interaction	Teacher-student interaction *It will positively impact the relationship between teachers and students* *In a typical learning system*, *we (students) do not get the chance to talk or interact with lecturer* *The lecturer individually takes care of the student*. *So*, *we can show our talent*
	Accommodating different learning styles	Learning style *The video lecture is suitable for visual & auditory learners* *I am a visual learner*. *I am using video lectures for biochemistry*

### Enablers

Enablers denote things that accelerate the flipped classroom implementation. The theme enablers are underpinned by several subthemes, including readiness, use of technology and available support, and current student-centred teaching learning practices (Table 3). Students were ready to engage in a flipped classroom as they are open to the idea *(“We are ready to face the flipped classroom”*) and they see the benefit of doing pre-class learning (*“Preparing ourselves before we go to the lecture will stimulate our thinking”)*. Considering the use of technology and available support, nursing students used smart devices to access their learning management system (*"we are using mobile phones and laptops to access the Moodle"*). Also, nursing students from university-C expressed the availability of technical support as *“If we have any problem with technology*, *we can call a special technical team here at any time”*.

Looking into current student-centred teaching learning practices, nursing students valued their learning experiences (*“We followed a problem-based learning approach*, *it helped me improve critical thinking and clinical skills"*). Teachers allowed students to clarify their doubts in face-to-face teaching (*“If we have any doubts*, *we can ask them during the session”*). Teachers had a practice of sharing their teaching material with students (*“Lecturers sometimes put their notes in the Moodle”*). Regarding current pre- and post-class activities, nursing students stated that *“some lecturers give information about upcoming session”* and *“most of the time after the lecture they (teachers) gave some assessment and assignment”*. Nursing students realized the importance of self-directed learning *(“Teacher gives basic information that is not enough”*).

### Challenges

Challenges require extra effort and determination to implement flipped classroom pedagogy. There were four main challenges identified in the FGDs: limited infrastructure, student motivation, lack of pre-class learning and training and development (Table 3). Not having access to a free internet connection (*“If we have free Wi-Fi*, *it will be better”*) and a lack of technical support (“*We did not get any technical support”*) were perceived as main infrastructure challenges, as reported by students from universities A & B.

Nursing students were also less motivated by passive learning experiences (*“We are like lazy in the lecture”*) and authoritative classroom management (*Teachers are being like authoritarian*, *like in school*). Nursing students reported their lack of pre-class learning practices (*“I’m going to lecture blindly”*) and expected to receive a training for enrolling into flipped classroom (*Training should need for enrolling this*). Also, they proposed some suggestions to develop the video lecture (*video should be attractive*) and strategies to enforce pre-learning practices (*“find some mechanism to see the video before coming to the face-to-face lecture”*).

### Benefits

Nursing students reported several benefits that they would expect to receive by engaging in a flipped classroom. Those benefits were categorised under six sub-themes: positive educational outcomes, knowledge transfer and application, flexible time management, flexible audio-visual material, and enhanced teacher-student interaction and accommodating different learning styles. In reference to the positive educational outcomes, nursing student stated that they would be more *“engaged”*, *“energetic”*, *“attentive”* and *“confident”* by enrolling into flipped classroom. Nursing students realised that the flipped classroom would enable them to apply learnt knowledge (*“In the flipped classroom*, *we will get the opportunity during the lecture time to apply the things what we learned*). They reported that the flipped classroom would also enable them to manage their time flexibly (*“we can watch the video lecture during our free time”*).

Nursing students understood the benefits of audio-visual learning material when incorporated into the flipped classroom (*“We can watch that according to our speed”*). They believed that flipped classroom pedagogy promotes teacher-students interaction (*“positively impact the relationship between teachers and students”*). Moreover, nursing students considered the flipped classroom was able to accommodate different learning styles (*“The video lecture is suitable for visual & auditory learners”*).

### Relationship between the identified themes

Relationships between the themes are explained as a developmental process in Fig 3. Enablers can maximise the benefits of the flipped classroom implementation. Challenges can be perceived as speed breakers that pose constraints for the developmental process but are indicators highlighting areas needing support. Benefits are definable outcomes that highlight the feasibility of implementing the flipped classroom pedagogy. Also, the perceived benefits could act as a catalyst to maximise the impact of the enablers and to address the challenges.

## Discussion

This mixed-methods study explored the feasibility of implementing a flipped classroom in the undergraduate nursing education in Sri Lanka from the students’ point of view. The study’s overall findings affirm that fostering flipped classroom practices in the above context is feasible. Moreover, the study revealed significant differences between the three universities for all domain measures. Even though the Sri Lankan government administers the three universities, there are differences between universities in terms of geographical location, grant allocation, and ranking. For example, University A and C are the highest ranked two universities in Sri Lanka. Therefore, further research is recommended to investigate why the perception of feasibility differs across the three universities.

The Sri Lankan nursing students, who were surveyed, expressed their personal and technological preference and readiness for enrolling in a flipped classroom programme. This might be due to student’s preference towards technology-enhanced learner-centred education. Certainly, the study cohort could be categorized as “Generation Z”, a unique and truly digital native generation [25]. Chicca & Shellenbarger [26] argued that Generation Z are frequent technology users and possess a reduced attention span, while desiring convenience and immediacy. Therefore, the flipped classroom would be an effective pedagogical option for connecting with Generation Z to increase attention span [26]. A Taiwanese study further supported this notion that undergraduate students (majoring in education) showed a high level of personal and technological preference and readiness for flipped classroom programmes [27].

In reviewing students’ views about current teaching-learning practice, they expected more application from classroom learning relevant to their clinical practice. Also, they requested an active and interesting instructional method. It is proposed that the flipped classroom is one of the most effective pedagogical methods for decreasing boredom [28]. Our findings indicate that students value exposure to student-centred learning approaches. This might be a gateway to flipped classroom pedagogy because evidence suggests that flipped classroom pedagogy emphasises student-centric teaching approaches [29].

Furthermore, the nursing students in this study understood the importance of pre-class learning and attending a face-to-face class with pre-existing knowledge. Nonetheless, pre-class preparation is seen as a significant challenge to flipped classroom implementation [30]. It is important that teachers find a way to approach and motivate students who are not completing pre-class activities. One way is to link pre-class activities with subsequent assessments. Therefore, completing pre-class activities can complement face-to-face teaching and add value to grade achievement [31].

Nonetheless, in this study, the majority of nursing students perceived the existing less-equipped learning environment in Sri Lanka may impede the successful implementation of the flipped classroom. Specifically, they were concerned about internet connectivity (e.g. free Wi-Fi) and technical support in the learning environment, which could apply to other developing nations and create a stumbling block for developing blended learning methods [32]. Interestingly, the nursing students who participated in the survey were eager to overcome the internet connectivity problem by using their personal devices and networks, suggesting a high level of interest in flipped classroom pedagogy. Moreover, the “Bring Your Own Device (BYOD) Model” showed increased productivity in terms of learning through utilisation of students’ own devices in the flipped classroom practice [33]. There is evidence to suggest that flipped classrooms can be effectively used even with limited technological provision. For example, pre-class learning materials can be stored in a DVD and given to students one week before the face-to-face class [1].

Educational videos are a crucial content-delivery tool in the pre-class phase of the flipped classroom pedagogy [34]. From the nursing student’s view, the educational video should be interactive to promote meaningful attention. Brame [35] affirms the view that the interactive feature of the video enhances student engagement and active learning. It is important to note that participants in this study expected hands-on training in the flipped classroom pedagogical system before enrolling in this new method.

In general, the nursing students in this study predicted positive educational outcomes or benefits that may result from flipped classroom pedagogy, namely improving learners’ engagement, enhancing attention, promoting self-learning, flexibility in learning, improving teacher-student interaction and providing them with the opportunity to apply learned concepts. There is ample evidence supporting students’ views that flipped classroom promotes a promising educational outcome in undergraduate nursing education [3].

These findings have several implications for teachers and university administrators. For the teachers, students appear to be less interested in passive learning inside the classroom. Therefore, it is vital to choose attractive and active teaching methods for in-class activities (such as team-based learning, problem-based learning, roleplay etc.) as well as providing pre-classroom materials. It is important to identify a meaningful mechanism to motivate students to complete pre-class activities. For example, when using educational videos in flipped classroom implementation, students prefer interactive videos. Some software enables teachers to develop simplified interactive videos, such as Camtasia Studio [36] EDpuzzle [37], Articulate storyline [38] and H5P [39].

For university administrators, students showed their readiness and interest in enrolling in a flipped classroom programme. Nevertheless, nursing students (e.g., from universities A & B) highlighted a lack of technological infrastructure in their learning environment, such as poor internet connection and limited troubleshooting. In addition, flipped classroom pedagogy is new to the students. It is worthwhile to conduct training and development courses for students about the basic concept of the flipped classroom and their roles in the teaching-learning process.

A limitation of this study is that the feasibility of implementing flipped classroom pedagogy was investigated purely from the students’ perspective. Even though students are indispensable stakeholders of the teaching-learning process, it would also be more encompassing to include the insights of teachers and university administrators in future research.

## Conclusion

This mixed-methods study explored the research question, *what is the feasibility of implementing flipped classroom pedagogy in the context of undergraduate nursing education in Sri Lanka from the perspective of undergraduate nursing students*? The findings revealed that nursing students expressed their personal and technological readiness and preference for enrolling in the flipped classroom programmes rather than traditional classroom-only programmes. However, technical resources need to be addressed so that up-to-date technology is available to enhance the flipped classroom pedagogical ideals. Nonetheless, the students in this study were ready to overcome technological inadequacy by using their personal devices and internet data. In addition, students highly valued student-centric pedagogical approaches, although training and development is required so that they can maximise the usage of such programmes. Moreover, the study also revealed significant differences in feasibilities between the three universities. These differences indicate that the needs and understandings of the flipped classroom model are not uniform across settings. Therefore, the unique characteristics of each university setting needs to be considered, e.g., technological readiness, to ensure that flipped classroom pedagogy enhances the teaching and learning within each university curriculum. In a future study, it is essential to explore the reasons for the feasibility differences between the three universities and to further incorporate teachers’ viewpoints to provide a more complete picture of the feasibility of flipped classroom pedagogy.

## Supporting information

S1 File Questions used in focus group interviews with students in Sri Lanka. (DOCX)

S2 File Data from questionnaire survey. (XLSX)
