# Supplementary material for: Assess the feasibility of flipped classroom pedagogy in undergraduate nursing education in Sri Lanka: A mixed-methods study
Source: PLoS One. 2021 Nov 5;16(11):e0259003. doi: 10.1371/journal.pone.0259003 (PMC8570468; doi:10.1371/journal.pone.0259003)
Supplement: S1 File — (DOCX) [file pone.0259003.s001.docx]

**Questions used in focus group discussions with students in Sri Lanka**

**Please tell me about your learning approach.**

*Possible follow-up questions:*

1. How are you utilizing technological device into your learning?
2. What is your preferred learning style (eg. verbal, visual. auditory…)?
3. What is your opinion about video lectures (or learning material in video format)?
4. How do you prepare yourself for attending a session?
5. How do you perceive your current face to face classroom activities?
6. How do you use Learning Management System (eg. Moodle or Blackboard) in your learning practice?
7. What is your opinion about team-based learning (learning with others)?

**What do you think about using a flipped classroom to support your learning?**

*Possible follow-up questions:*

1. How do you feel that the flipped classroom impacts your learning?
2. Will it change the way you learn? Positively and/or negatively?
3. How does using flipped pedagogy work with your learning?
4. What extend you feel flipped class impacts your educational outcomes?
5. How do you feel that the flipped pedagogy impacts your relationship with teachers?

**What impacts your ability to use flipped classroom?**

*Possible follow-up questions:*

1. How do you feel that the available resources in your institution which affect your ability to use flipped classroom?
2. How does technical support (available in your institution) which impact your ability to use flipped classroom?
3. How do you feel that flipped pedagogy impact your time?
4. How valuable do you think flipped classroom is?
5. How is your readiness to receive the flipped classroom sessions?
6. What kind of training necessary for you to adopt the flipped learning?

**Is there a question I haven’t asked? Is there anything else you want to tell me?**
